# Implementing a Digital Child Behavioral Health Prevention Program in Faith-Based Settings in Uganda: A Feasibility Study

**DOI:** 10.18103/mra.v12i10.5926

**Published:** 2024-10-30

**Authors:** Keng-Yen Huang, Janet Nakigudde, Tusiime Christine, Sabrina Cheng, Dickson Muyomba, Eddie Tinka Mugisa, Elizabeth Nsamba Kisakye, Hafsa Sentongo, Antoinette Schoenthaler, Omar El-Shahawy, Devin Mann

**Affiliations:** 1 Department of Population Health, New York University Grossman School of Medicine, 180 Madison Ave, New York, NY 10016, USA.; 2 College of Health Science, Makerere University, P.O. Box 7072, Kampala, Uganda.; 3 Uganda Ministry of Education and Sports, Kampala, Uganda; 4 Uganda Ministry of Health, Plot 6 Lourdel Road, PO Box 7272, Kampala, Uganda; 5 Institute for Excellence in Health Equity, New York University Langone Health, 180 Madison Ave, New York, NY 10016, USA; 6 Department of Global and Environmental Health, NYU School of Global Public Health, 708 Broadway, New York, NY 10003, USA; 7 MCIT Department of Health Informatics, New York University School of Medicine, 1 Park Ave, New York, NY 10016, USA

**Keywords:** Faith-based-organization, parenting, health literacy, mHealth

## Abstract

**Background:**

The burden of pediatric mental disorders in low-and middle-income countries (LMICs) is tremendous, but solutions for addressing the burden remain limited. Although digital solutions have potential to improve prevention services, such solutions have not been systematically tested in these countries.

**Objective:**

This study explores the use of a digital parenting intervention tool designed for pediatric behavioral health, known as the Pediatric-Behavioral Health Digital Tool, in a preventive service model for low resource communities. We study the feasibility of implementing this new digital health service model and preliminary estimate the potential impacts on parenting and child social emotional outcomes when the program is implemented in faith-based organizations in Uganda. The Pediatric-Behavioral Health Digital Tool is a preventive intervention designed to be implemented by trained community-health-workers to facilitate caregivers’ access to the preventive mental health service in community for their young children. The tool is based on the screening, brief intervention, and referral to treatment prevention service model for promoting pediatric behavioral and mental health.

**Methods:**

The evaluation study was designed using a pre-post assessment design. The content in Pediatric-Behavioral Health Digital Tool was co-designed with local expert and iteratively adapted based on parents and caregivers as well as community-health-workers and experts who were invited to provide their feedback and suggestions for improvements in content, functions, and delivery model through a series of focus groups and workshops. This pilot evaluation focuses on the pre-post changes of the intervention families (91 families) and 10 community-health-workers.

**Results:**

We found high acceptability, appropriateness, and usefulness of the program based on the intervention families’ community-health-workers’ report. Intervention parents felt safe in using the digital toolkit. Parents felt comfortable for the CHWs asked them personal questions. In estimating the impacts, we found some expected findings on parenting and child social emotional health. Specifically, we found intervention parents become more mindful in their parenting (d=1.61, p=.049), and felt more effective in discipline their child’s misbehavior (d=1.29, p=.003) after they receive the intervention. For children, we found improvement on children’s social emotional outcomes, measured by decreased parent-child conflict (d=−1.08, p=.002) and increased child emotional regulation skills (d=1.0, p=.049) after their parents receive the intervention.

**Conclusions:**

Our Pediatric-Behavioral Health Digital Tool has potential to provide a cost-effective service solution to provide preventive mental health care in communities to promote child social-emotional and mental wellbeing in low-resource settings.

## Introduction

Mental, neurological, and substance (MNS) disorders is highly prevented, account for 10–14% of the Global Burden of Disease^1,2^. MNS burden is even greater in low-and-middle income countries (LMICs) because of the high living adversity (e.g., high poverty, violence, health problems), and poor health care systems^3,4^. Low-cost preventive solutions for reducing burden for LMICs in communities is urgently needed. To address the preventive service and evidence-based intervention gaps, we aim to develop and test a technology-supported population approach of child mental health promotion to be used in community settings. We have developed a digital health tool—the Pediatric Behavioral Health Digital Toolkit (P-BHDT) and implemented it in community-based organizations to enhance accessibility and acceptability. The goal of this paper is to report the design of the P-BHDT and the feasibility of implementing the P-BHDT program in faith-based organizations (FBOs) in one LMIC-Uganda.

### RATIONALE FOR PROVIDING P-BHDT PREVENTIVE PEDIATRIC BEHAVIORAL HEALTH SERVICE IN FAITH-BASED ORGANIZATIONS (FBOs) IN LMICs

Our decision to provide the P-BHDT program and preventive service in FBOs is guided by three areas of evidence for promoting population health in low resource settings.

#### One, a body of health research suggests that FBOs are ideal setting for providing a low-cost task-shifting approach of public health Interventions to improve population health in Sub-Saharan African (SSA) countries^5^, which can also be applied to the MNS burden management.

Supporting evidence includes: **(i)** FBOs have successfully run health programs using task-shifting approach for prevention, screening and treatment for highly stigmatized HIV/AIDS problems in SSA countries^6–10^, and an emerging literature in developed countries has documented the effectiveness of psychosocial interventions in improving FBO members’ health knowledge and awareness^11^; **(ii)** the central role of FBO in SSA families’ religious, social and cultural life, and as a source of health support^12^. A large percentage of SSA families attend activities in FBOs, and often rely on these informal sources of support from FBOs to manage psychological problems^11^; **(iii)** FBOs provide an effective means in communicating faith and other health messages to a larger population,^13^; **(iv)** majority of FBOs in SSA countries have health service structures and provide health services to members, which are generally organized by the FBO health committees and voluntary CHWs^14^; and **(v)** FBOs’ mission is to increase their members’ awareness of social issues (including health, parenting) and work *to advocate health for all*^15^. *For our targeted country, Uganda, 99% families attend activities in FBO, and the Uganda Catholic Medical Bureau provides about 50% of the health services in the country*^12,16^.

#### Two, health service research has shown that providing screening, health literacy, and early referral/intervention in community settings as a good evidence-based clinical practice guideline for managing and controlling burden of MNS disorders.

Numerous scholars and international institutions have suggested integrating behavioral health **s**creening, **b**rief intervention, and **r**eferral to **t**reatment (SBIRT) in routine primary care as an effective approach to promote population health^17–20^. To provide SBIRT service set-up, several collaborative models have been tested in developed countries and have demonstrated effectiveness in overcoming economic and structural barriers, and improving service access, quality of care, and patients’ mental health^19,21–26^. Integrating SBIRT service in service organizations requires setting up 5 core components: **1)** provider education, **2)** a standardized assessment tool for families, **3)** a health coordinator to assist in collection and collation of the assessment components, **4)** health literacy materials for families, and **5)** referral pathways to mental health care, and other resources^27^. Given the similar structures in FBO health committee and service setting^14^, and the potential that SBIRT model may improve access, child behavioral health, and curtailed costs, it is critical to test the feasibility of the SBIRT model in FBOs in SSA.

#### Three, rapid growth of digital health provides new solutions to address service access and implementation barriers.

The rapid advancement of digital health and trial evidence for mobile approaches of behavioral health interventions in developed countries have provided new solutions for providing accessible preventive behavioral health services^28,29^. Digital health holds many advantages, including providing low-cost personalized support services, improving distance communication barriers, and providing an easier and sustainable implementation model^28,30^. Digital approach has shown to be effective in promoting child and family wellbeing, improving pediatric behavioral health services in primary cares and communities in high-income countries^31^. Our goal is to adapt the *SBIRT to an e-SBIRT pediatric behavioral health preventive* service model and provide the digital service in FBOs.

### THE PEDIATRIC-BEHAVIORAL HEALTH DIGITAL TOOL SOLUTION

The P-BHDT is designed to address three critical areas of child mental health needs in LMICs. We aim to address:

- *Children’s preventive behavioral health needs* by providing early screen to families to promote awareness and early intervention.
- *Parenťs/caregivers’ needs for access to parenting knowledge and evidence-based strategies* by integrating pediatric behavioral health literacy information in the P-BHDT.
- *Service access and community capacity gaps* in providing services by training community-health workers (CHWs) in FBOs to provide preventive behavioral health service.

### IMPLEMENTATION OF THE PEDIATRIC-BEHAVIORAL HEALTH DIGITAL TOOL PREVENTION PROGRAM (WHERE, WHO, AND HOW)

The P-BHDT, an e-SBIRT preventive behavioral health service model, is designed to be implemented by (CHWs) in community settings. Given that FBO is the centerpiece of SSA families’ social and cultural life, and a source of health support [13], we leverage the existing FBO structure and practices, and train FBO-health team members and CHWs to provide P-BHDT in FBOs. In addition, considering the high proportion of parents in LMICs with low literacy and limited experience with technology-based services, training CHWs to facilitate and support parents in use of the digital toolkit is critical. The P-BHDT content was co-designed with local experts and iteratively adapted based on feedback from parents, caregivers, CHWs, and experts. These stakeholders contributed to improvements in content, functions, and the delivery model through focus groups and workshops. This collaborative design process not only facilitates a smoother implementation process by addressing potential mistrust and conflicts among stakeholders, but also ensures the integration of the P-BHDT into existing systems by respecting and considering the sociocultural contexts

**Figure 1.**
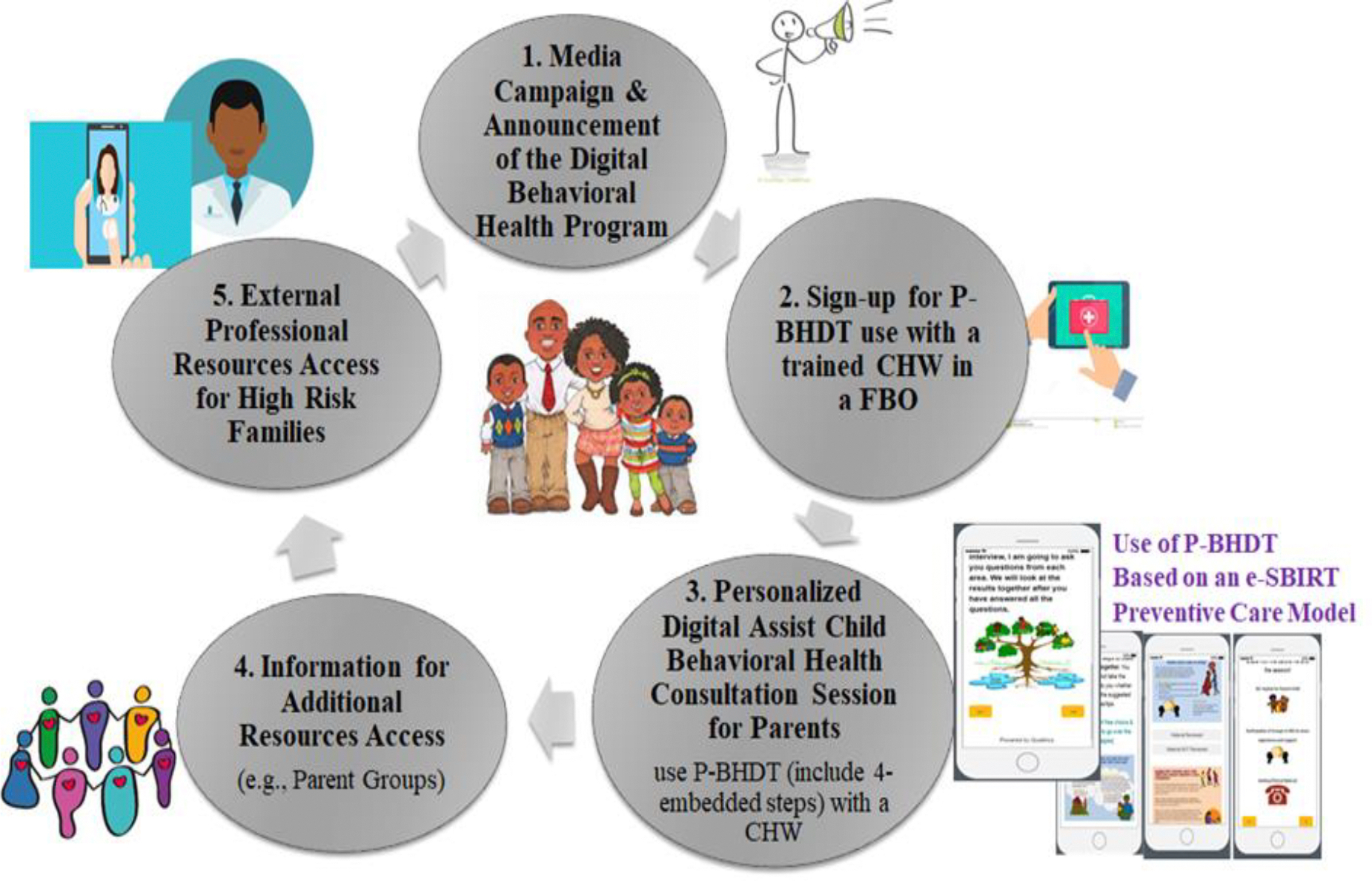
Process for Implementing the P-BHDT supported e-SBIRT Preventive Care in FBOs

Figure 1 shows the processes to implement P-BHDT and e-SBIRT service model. To ensure high engagement from the targeted users, the trained CHWs were encouraged to work with FBO leaders to develop announcement strategies to share the service information to eligible families. A brief service information video was also created to share with families. After families signed-up for the Toolkit session, the trained CHWs arranged a time to meet with parents to use the Toolkit and provide needed support. Parents follows a 4-step procedure included in the P-BHDT, which typically lasts for 1–2 hours: (1) Parents first answered questions from a set of standardized tools that screen children’s behavioral health and related family risks^20,27^, (2) After the screening, parents reviewed a tailored report (generated by the toolkit) that highlight their strengths and weaknesses, and reflect areas for making changes; (3) Parents then reviewed child mental health literacy materials generated from the Toolkit based on screening results, and (4) For high-risk families, parents received support resources information (i.e., parenting program information) or were referred to external professional resources. This initial version of the P-BHDT Toolkit focuses on early childhood, and targets parents or primary caregivers of 3 to 8 years old children. For this pilot study, the Toolkit was developed as a Single Session Intervention (SSI) using Qualtrics software, available in the offline version. The SSI approach was chosen for its flexibility and cost-effectiveness, making it particularly beneficial for implementation in FBOs within LMICs. Single session intervention provided as-needed treatment that can be completed once, repeated multiple times, or used as an adjunct to longer-term care, making it a versatile tool for addressing mental health needs in resource-limited settings. Our long-term goal is to develop an App that can be implemented by CHWs to follow up with families or as a self-managed App for higher literacy parents.

### STUDY OBJECTIVES

This paper has two objectives.

1. To assess the feasibility of implementing the P-BHDT preventive program in FBOs in low resource LMIC communities.
2. To preliminary estimate the impacts of the P-BHDT prevention program on parenting and young children’s social emotional outcomes.

## Methods

### OVERVIEW OF THE STUDY DESIGN

This study aimed to evaluate feasibility of implementing P-BHDT program (an e-SBIRT service model implemented by CHWs) in FBOs in Uganda and preliminary estimate the impacts on parenting and child social emotional outcomes. To evaluate the *implementation feasibility*, three areas of feasibility indicators were examined: (i) acceptability, appropriateness, and usefulness of the P-BHDT (i.e., contents and service model); (ii) users’ comfort and trust to the implementation procedures (i.e., comfort in answering personal questions; trust data security/safety); and (iii) FBOs’ ability in implementing the P-BHDT preventive service model (i.e., CHW competency, ability in engaging and supporting families).

**Figure 2.**
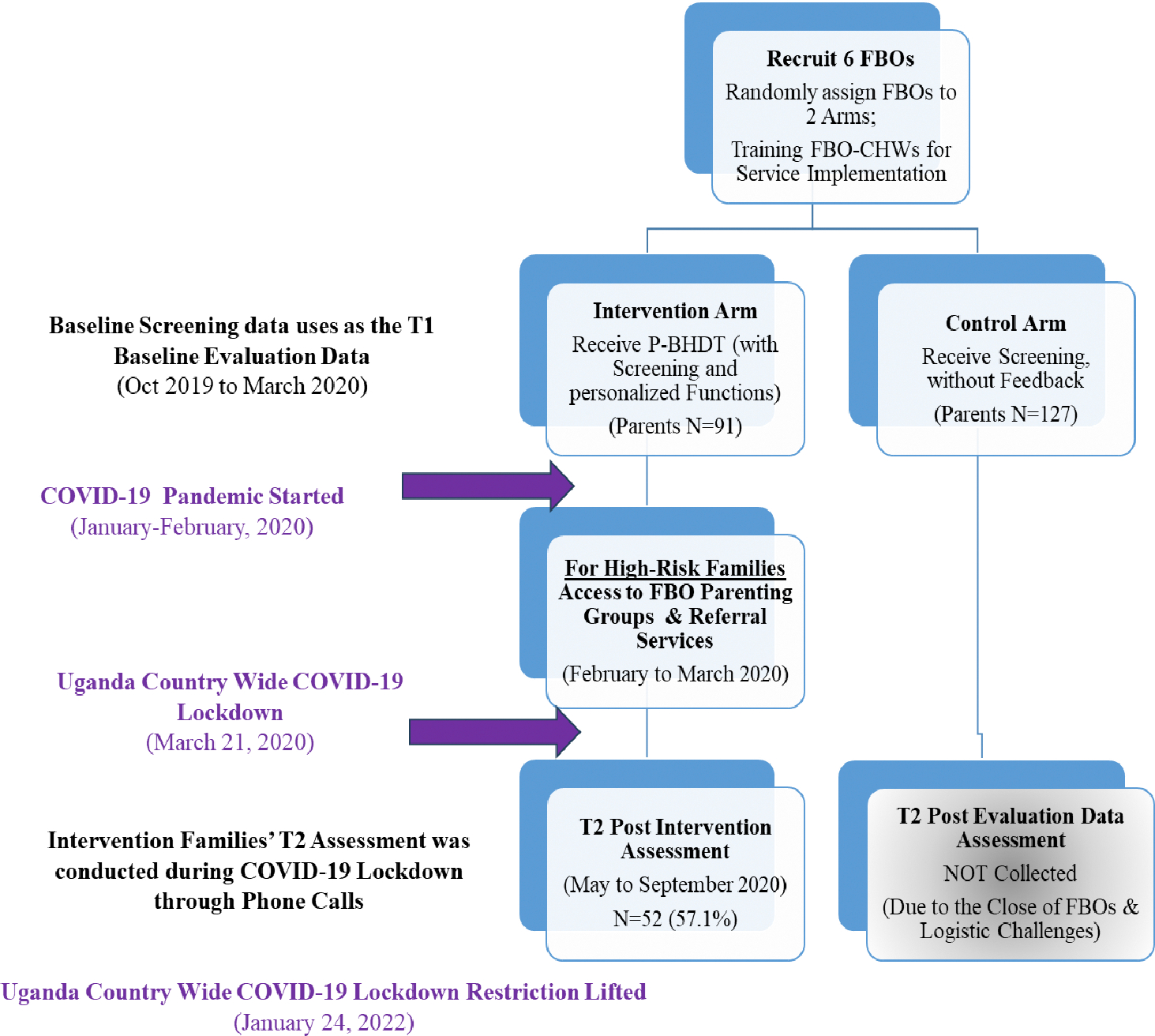
Consort Diagram for the Pilot Feasibility Trial Study (in the COVID-19 Pandemic Context) ***Note.*** The preliminary estimation of the intervention effectiveness is based on the intervention sample only because of the delay of the data collection for the control sample. T2 data for the control sample was not collected till the end of the COVID-19 pandemic (around October-November 2021, about 1 year apart from the intervention families’ T2 data collection time).

To *estimate preliminary effectiveness*, we had planned to apply a cluster randomized controlled trial (cRCT). Due to the COVID-19 pandemic, we changed to a pre-post assessment design. Figure 2 Consort-diagram provides the context of the original trial design and evaluation timeline. To provide a better context for the sample recruitment procedure, and pre-pandemic activities, we provide a brief overview of the original design here. The study was initiated on October 19, right before the pandemic. The original cRCT include 6 Ugandan FBOs (Christian-based). In the three FBOs randomized to intervention, CHWs were trained to implement P-HBDT program to families with 3–8 years old. For the FBOs randomized to control, CHWs were trained to only provide screening using the digital tool but without any tailored report or feedback for families. Families signed up to the study were assessed at baseline and post-intervention (about 5–6 months after baseline). Two areas of outcomes were assessed: (i) parenting outcomes (i.e., mindfulness, effectiveness in discipline, setting rules, nurturing parenting); and (ii) children’s social emotional outcomes (i.e., emotion regulation, social relationships).

After Ugandan government enforce the country-wide COVID-19 lockdown on March 21, 2020, we stopped all the activities because FBOs were closed. Fortunately, most of the intervention activities were completed before the country-wide lockdown (conducted between Oct 2019 and February 2020). To capture the impact of the intervention, we prioritized intervention families and used phone interviews to conduct Time 2/post-intervention assessment (carried out between May and August 2020, in the early phase of COVID-19). We were able to interview about 57% of families. Many families were not able to reach because of relocation (from urban to village during the lockdown). We were unable to reach most control families; thus, the Time 2 assessment for the control families were not conducted till the lift of the country wide lockdown, which is in January 2022 (about 2 years after the 1^st^ assessment). Given this context, for this feasibility study, estimation of the preliminary effectiveness was based on the intervention sample only.

### PARTICIPANTS AND PROCEDURES

The FBOs were selected based on recommendation from our research staff outreach and Advisory Board members. The FBO leaders who were interested in having his/her FBO participate in the study were enrolled. Six FBOs (or 3 match-paired similar in size and location within each pair) were recruited. Within each pair, we randomly assigned one to intervention and one to wait-list control. The CHWs who were recommended by the FBO leaders and interested in participating in the study (3–4 per FBO) were consented. Participating CHWs from all FBOs receive 1-day training on family engagement and using e-screening. The trained CHWs then support the recruitment and baseline e-screening with families. The CHWs from intervention FBOs receive an additional 3-day training on using and implementing P-BHDT. They then implemented the P-BHDT program to parents. During the first 2 months of implementation, they also received 4 group-coaching sessions from Ugandan mental health experts. The CHWs and caregivers from the wait-list control FBOs received intervention training after the completion of the evaluation study.

To recruit parents, the research team and CHWs worked together to provide two information and recruitment sessions at each FBO. Caregivers who had 3–8 years old children and interested in the intervention were consented. After consenting, they scheduled a session with CHWs to utilize P-BHDT. For the evaluation, only one parent per family was allowed to participate. For representation, we recruited 30–40 families from each FBO.

### STUDY MEASURES

The evaluation was guided by the Proctor’s implementation outcome framework^32^. During the consent process, parents completed a demographic survey, FBO leaders completed a FBO Organizational Questionnaire, and CHWs completed a demographic along with a technology use and readiness survey^33^ (9 items, alpha=.72; e.g., I usually do well using digital devices; I have interested in working with health related digital tool). Below, we describe measures used to assess implementation feasibility and estimate effectiveness outcomes. Where possible, we employed measures with demonstrated reliability and validity from our previous studies in Uganda^34^. Psychometric properties using data from the current sample are reported below.

#### Implementation Feasibility Measures.

To assess implementation feasibility, we applied the Acceptability, Appropriateness; and Usefulness scales developed by Weiner et al.^35^. Additional process feasibility measures (i.e., trust, comfort level) and CHWs ability in implementing the program were developed by our team. Table 2 provides measurement constructs, example items, and scale reliability info based on our study sample. These measures were used for the intervention sample only, and completed by CHWs and parents after the P-BHDT program (about 3 months after the baseline). For parent P-BHDT users, they were also asked to complete five exit questions to share their experience and feedback in toolkit use (See Figure 4 for the items).

#### Family Outcome Measures.

To assess parenting outcomes, three parental self-report measures were used to assess 5 different areas of parenting. *The Parenting Strategies Questionnaire (PSQ)* assesses the frequency in which parents apply a range of evidence-based behavioral management strategies (e.g., praise, proactive strategies) and discipline practices at home on a 5-point scale (1= never, 5= very often). Three subscales, *Nurturing Parenting* (9 items, α = .78), *Setting Clear Rules/Routine* (4 items, α = .69), and *Effectiveness in Discipline* (2 items, α = .66)^36^) were applied. The Mindfulness in Parenting questionnaire assesses mindful parenting and parenťs ability to maintain awareness when managing child behavior. The mindful discipline subscale was adapted to measure parenťs discplinary intentionality on a 4-point likert scale (1=Infrequently; 4=almost always)^37^. The Patient Health Questionnaire-4 (PHQ-4; α = .76) was applied to assess parental mental health, which is an ultra-brief parent-report scale (included 2 anxiety and 2 depression items). The scale has been widely used and validated in many countries [49–52]. Parents rated symptoms on a 4-point scale (0 = not at all; 3 = nearly every day). The PHQ-4 total sum score and mental health problems binary variable (PHQ-4 score ≥ 6) were created.

To assess child outcomes, two measures were used. *The Social Competence Scale* [66, 67] assesses children’s social emotional skills. We apply the Emotion Regulation subscale (6 items, α = .78) in this study. Parents were asked to rate how well the statements described their child on a 5-point scale (0 = not at all to 4 = very well). To assess children’s social relationship with parents, the *Conflicted Parent-Child Relationship* scale was applied (5 items; α = .85; e.g., child and I always seem to be struggling with each other) [64]. Parents rated child-parent relationship on a 5-point scale (1 = strongly disagree; 5 = strongly agree).

Both parenting and child outcomes were assessed at baseline (T1) and post-intervention (T2). For parent-reported measures, assessments were completed in English and/or Luganda.

### ANALYTICAL APPROACH

To examine implementation outcomes, we carried out a series of descriptive analyses for the fidelity indicators using the intervention data. To preliminary estimate effectiveness outcomes on parenting and child outcomes, we focused on the intervention sample and estimated pre-post changes. We did not include control families because of the change of the design due to the disruption of the COVID-19 pandemic (see Method above). Outcome estimation was evaluated with a multivariate analysis of variance-type analysis using linear mixed effect models (using SAS PROC MIXED)^38,39^. We modeled pre-post trajectory changes for parenting and child outcomes (constructs listed in Table 3).

To consider partial missing data for the intervention sample, we inspected missing data patterns. We found that parents with and without follow-up data did not differ on baseline family demographic characteristics (i.e., education, sex, religion, employed status, food insecurity) and levels of parenting or child outcomes (listed in Table 1). Therefore, we assumed data were missing completely at random. To account for missing data, we applied a multiple imputation strategy, in which analyses were replicated in 10 imputed datasets. The final inference was derived by combining the results using the SAS PROC MIANALYZE procedure^39^.

## Results

### BASELINE CHARACTERISTICS

#### Characteristics of FBOs (inner setting).

As shown in Table 1, FBOs had an average of 300 congregation members (ranging from 70 to 480). Demographic data collected from FBO leaders showed that 100% of FBOs provided children’s groups, 75% provided child development or positive behavior promotion program. FBO leaders also reported that most challenges faced by children were lack of parental support (i.e., parenting reliance of house helper, lack of safety monitoring), family poverty (i.e., difficulties paying school fee), and experiencing family adversity (polygamy, domestic violence, single parenthood), poor education of parents.

#### Characteristics of CHWs.

**Figure 3.**
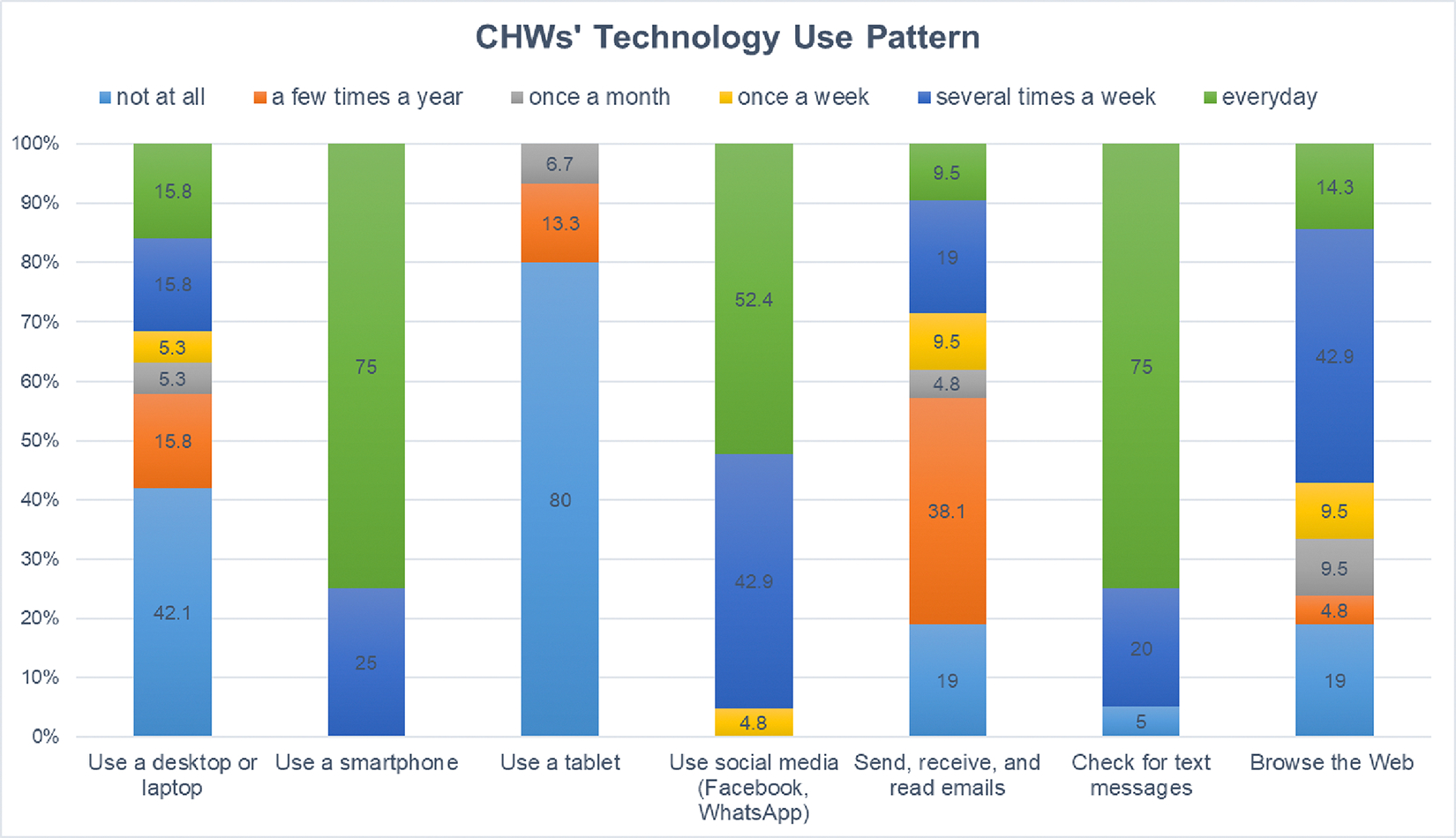
CHWs’ Technology use pattern ***Note.*** numbers represent % of CHWs endorse the response. Results were based on all CHWs (n=21) recruited from intervention and control FBOs, with similar patterns for intervention and control CHWs.

On average, CHWs had served/worked 11.04 years in FBOs and about half of CHWs have children themselves. Only a small percentage of CHWs have been involved in any child health promotion programs before (15.8%). The majority of the CHWs had a smart phone (81%). Figure 3 shows CHWs technology use patterns. We found most were not using tablet (80%), the tool that we used to implement P-BHDT. We also found that most CHWs used social media regularly, such as Facebook or WhatsApp (95% use several times a week or more). Less than half were using desktop or laptop regularly. Technology readiness for CHWs were relatively high (Mean (SD)=4.19 (.41)).

#### Characteristics of Families.

Most participating family members were female caregivers (82.5%), and 60.3% were mothers. About 70.1% caregivers were married or live with their partner, 70% were employed, 18.8% experienced food insecurity, and 36.1% had high school or more education.

### IMPLEMENTATION FEASIBILITY OUTCOMES (IN INTERVENTION FBOs)

#### Feasibility: Acceptability, Appropriateness, and Usefulness.

Table 2 shows that both parent (P) and CHW report high acceptability, appropriateness, and usefulness of the Digital Toolkit Program, based on the rating collected 2–3 months after the P-BHDT program.

**Figure 4.**
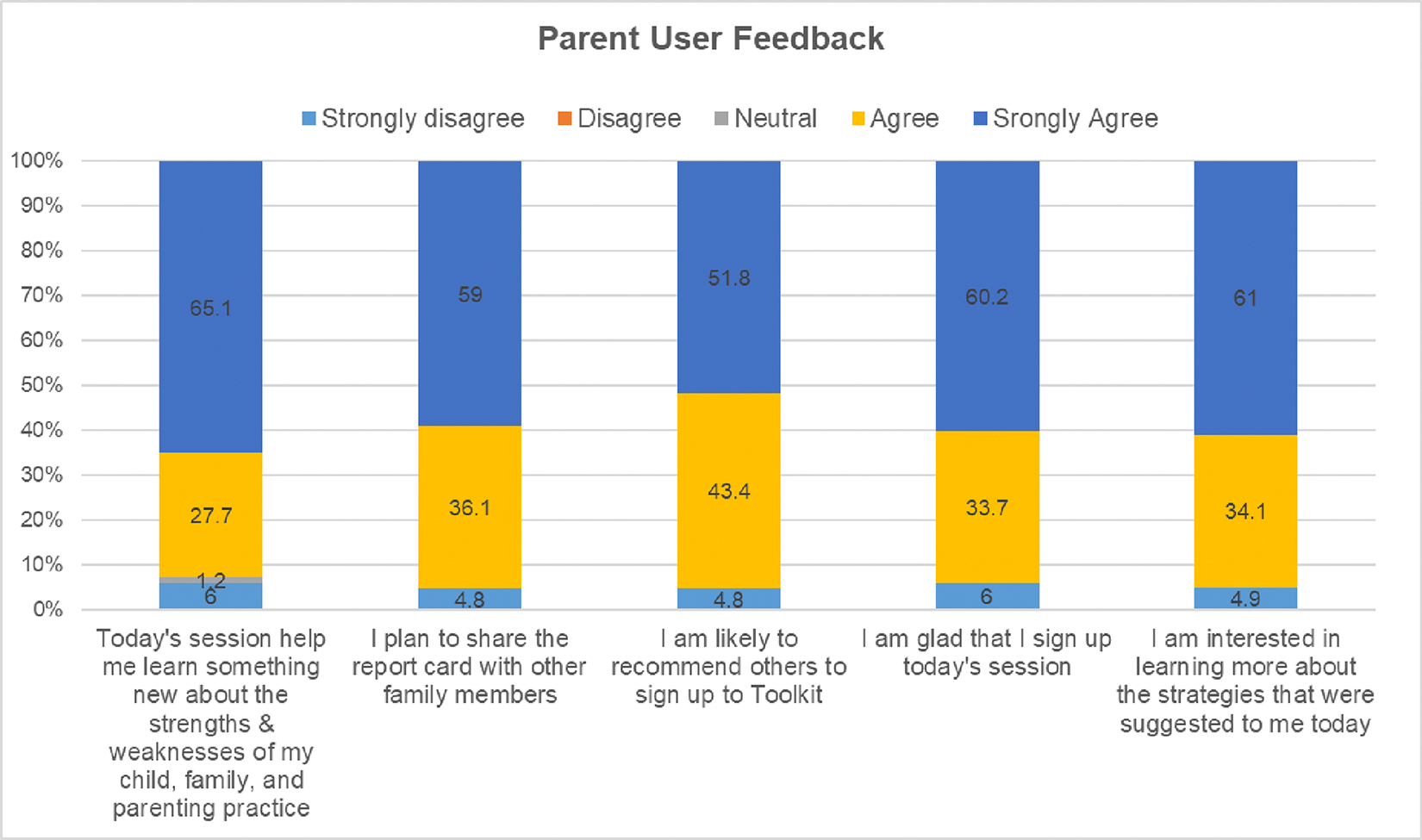
Users’ Experience and Feedback (Immediate After the Toolkit Use) ***Note.*** 91% mHealth users provide feedback, and 92.8% to 95.2% rated Agree or Strongly Agree on the items.

Implementation feedback gathered from intervention parents at the end of the P-BHDT session was analyzed to further understand parents’ experience and perceive usefulness of the session. We found that most parents (>90%) agree or strongly agree that the session helped them learn about their families’ strengths and weaknesses, glad that they signed up the session with CHWs, plan to share the results with other family members, and recommend others to sign up to use the toolkit (see Figure 4).

#### Feasibility: Implementation Procedure.

To assess the feasibility of the digital toolkit implementation, we assess users’ Comfort, trust, and experience (Table 2). We found parents trust the security and safety of the data, and they also felt comfortable in answering personal questions when CHW asked. The CHWs also perceive high feasibility to implement the procedure of the P-BHDT program. Mean scores on the feasibility rating were all > 4.0 on a 1–5 point Likert scale. In addition, for those been recommended to use the parenting program, 72% of parents used the recommended service (i.e., parenting groups) after the toolkit use.

#### Feasibility: CHWs’ Ability in Implementing the P-HBDT Program in FBOs.

**Figure 5.**
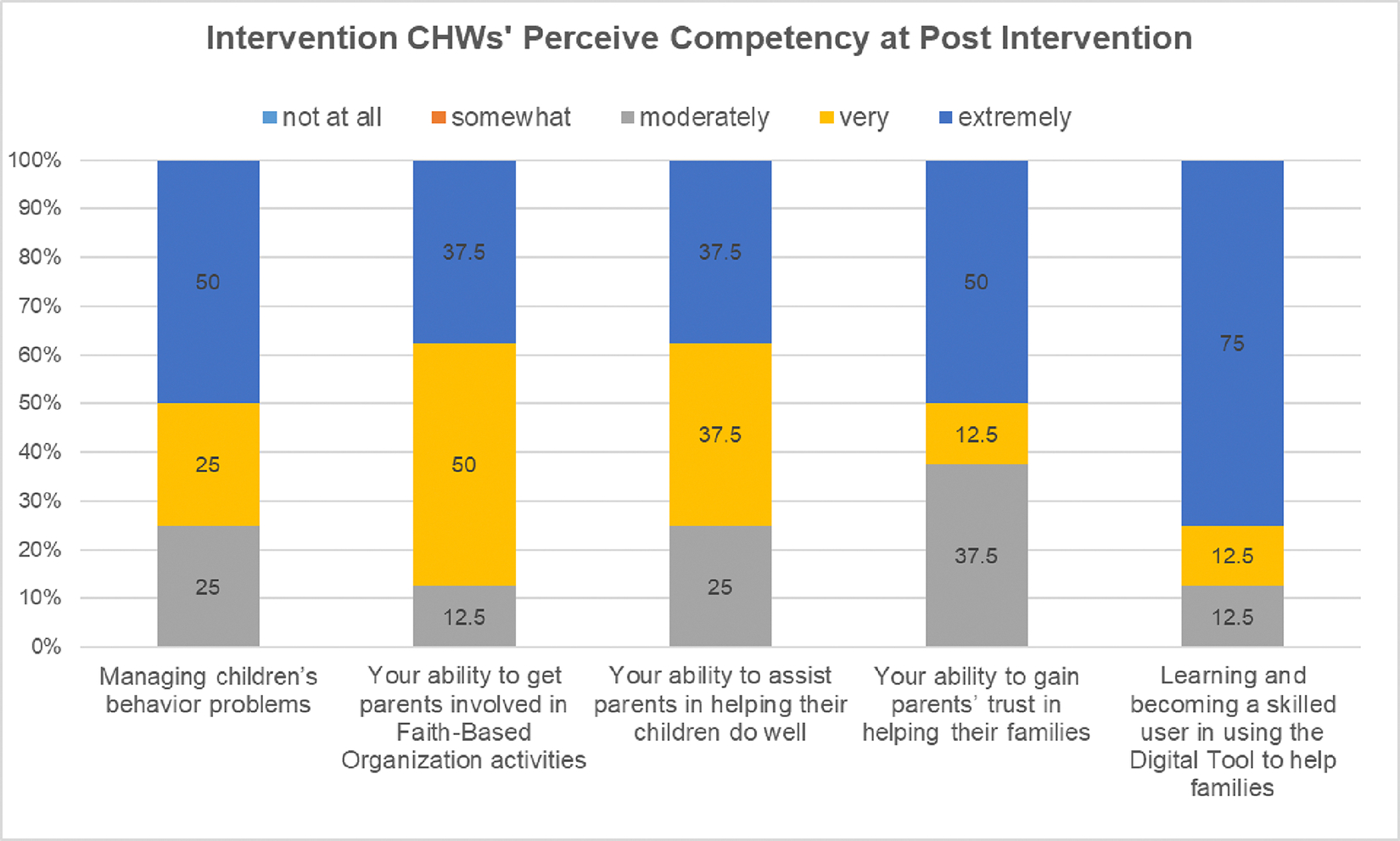
Intervention CHWs’ Rating of Their Competency (How confident are you in ….) ***Note.*** The data were provided by intervention CHWs after the Toolkit Program Implementation.

Parents shared that CHWs were highly competent in supporting them in using the digital toolkit (Table 2). Trained CHWs also self-rated high competency in their ability in providing P-BHDT program. More than half of CHWs self-rated very or extremely competent in their ability in becoming skill in using digital tool to help families, in gaining parents’ trust, in assist parents in helping their children do well, in involving parents, and in managing children’s problems (see Figure 5).

### PRELIMINARY EFFECTIVENESS EVIDENCE OF THE P-BHDT PROGRAM (THE e-SBIRT SERVICE MODEL IN FBOs).

Pre-post assessment of parenting and child outcomes (based on the imputed data) found some expected outcomes (see Table 3).

#### Parenting Outcomes.

In parenting domains, we found that intervention parents significantly improve in their mindfulness parenting (Cohen’s d (d) = 1.61), and perception of their effectiveness in discipline/managing their children’s misbehavior (Cohen’s d (d) = 1.29) after their participation of the P-BHDT program. The effects on changing parenting behaviors were lower, with small to moderate effect on setting-up routine and applying nurturing parenting strategies (such as praise or use supportive strategies), with *ds* range .27-.31. Somewhat unexpected, we found a significant increase in parental mental health problems, measured by PHQ4. The increase of parental mental health problems might reflect stress related to COVID-19 pandemic.

#### Child Outcomes.

We found significant improvement in children’s emotion regulation (Cohen’s *d (d)* = 1.09) after parents received P-BHDT. Similarly, we found significant improvement on parent-child relationships, indicated by reduction of parent-child conflict relationship (Cohen’s *d (d)* = −1.08).

## Discussion

To our knowledge, this is the first study to test whether an e-SBIRT health service model can be applied to child mental health prevention through digital solution and community-based organizations to promote child population behavioral and mental health. Overall, findings suggest the feasibility of implementing e-SBIRT model in FBOs by CHWs with the digital tool P-MHDT support, and the potential of using this digital service model to address multiple prevention service gaps and families’ needs. Our study found that P-BHDT program resulted in large effects in improving parents’ mindfulness parenting and perceived effectiveness in discipline their child’s misbehavior. We also found large effects in improving children’s emotion regulation skills and child-parent relationship (reduction of conflict relationship). Although the impact on parenting behavioral practice (e.g., use of nurturing parenting strategies, setting rules/routine) by parent-report did not reach statistical significance, the effect size was meaningful (d=.27-.31) and similar to other traditional caregiver/parenting intervention and in the desire direction^40,41^. To further understand whether the improvement in parenting was associated with improvement in children’s outcomes, we carried out post-hoc analyses (by examining changing scores and their association). As hypothesized, we found significant positive associations between improved parenting and improved child outcomes, suggesting a possible mediational link that can be further examined in future larger scale studies. Preliminary effectiveness findings suggest that our approach not only promotes parent awareness, parenting, but also promotes parents’ child behavioral health literacy.

Previous pediatric behavioral health research has tested SBIRT in primary care settings through provider staff, but not in FBO settings and by CHWs in LMICs. We extended the SBIRT to an e-SBIRT preventive behavioral health service model to communities in Uganda. Our P-BHDT program introduced Ugandan communities to an early screening and early intervention concept. CHWs from FBOs were trained and then provided screening, health literacy, and early referral/intervention to their congregational families, which strengthens FBO capacity and empowers FBOs to provide accessible preventive behavioral health services that benefits their congregation members and communities. Our feasibility data from CHWs and parents reveal that the implementation approach was highly acceptable, appropriate, and useful. Our study adds new evidence for a low-cost approach to provide a digital and personalized pediatric behavioral health prevention program in low-resource communities. Findings also suggest the potential to apply and scale the P-BHDT model to other low resource communities/countries to address child mental health needs and service needs. However, it is important to consider the local context and work closely with local stakeholders to ensure buy-in, and cultural appropriateness and relevance.

From research design to the implementation we utilized a collaborative process. We engaged diverse stakeholders to ensure the P-BHDT met the needs of the community. This context-specific approach was a key factor in the success of the intervention. From the users’ (parents’) and providers’ (CHWs’) digital engagement perspective, we considered their digital literacy and usage habits, and integrate a few strategies in the implementation design. *For parents*, we considered parents’ education, digital health literacy, and trust factors. Considering a high proportion of Ugandan parents has lower than high school education and new to digital health model, our experiences suggest that offering CHWs’ support in the implementation process is critical. The existing relationships of the CHWs with the community and families also likely facilitated the acceptance and use of P-BHDT. A self-management approach of digital health intervention can only be relevant to high-literacy parents. Also, given the central role faith and religions plays in shaping mental health perception and parenting practice in Uganda, we embedded the P-BHDT in FBOs. We also consider time limitation and access, allowing users to use P-BHDT once or multiple times. To promote trust, we worked with FBO leaders to develop announcement/messages. We found that FBOs’ leaders’ support and endorsement of the P-BHDT program facilitated high parent participation rate, especially in utilizing parenting groups facilitated by their CHWs after the toolkit use (i.e., 75% utilization rate). Users (parents) also felt comfortable in discussing personal questions with CHWs, perceived their CHWs with competency and ability to provide the program, and trusted the data security. *Similarly, for implementers (CHWs)*, we considered their digital readiness and usage patterns when designing the implementation procedure. The measurement that we applied (in assessing digital readiness at baseline) may also be applied to other digital implementation research in planning for digital health training. Many digital interventions in LMIC fails because 1) they do not fully engage with the community context 2) involve the community only after the intervention has been designed and implemented 3) exclude key players or users of the intervention, and 4) lack innovation during the implementation process^42^.

Between October 2019 and November 2021, during the course of this study, Ugandan families faced significant challenges due to prolonged COVID-19 lockdowns, school closures, political tensions, and economic hardships. Of note, there was a significant increase in parental mental health challenge. This finding was consistent with other research documented rising mental health challenges in global COVID-19 pandemic context^43,44^. Despite the negative impact of pandemic on parental mental health, our study demonstrates positive results on parenting and child outcomes. Findings suggest the potential of using the e-SBIRT/P-BHDT service model to address children’s mental health needs in crisis contexts. As digital intervention grows in LMIC and the use of smartphones become increasing common, it is also worth exploring other digital strategies to integrate parental mental health intervention in our digital program to maximize the benefit in the crisis context. Telecommunication format can potentially offer families with more flexibility in how they access, engage, and share the materials with their community.

### STUDY LIMITATIONS

This study has several limitations. *One*, this is not an RCT study. Although our original intention was to apply an RCT design, the disruption of COVID-19 pandemic impacted this. Future study should further investigate the short and long-term impacts using an RCT evaluation design. *Two*, although the impacts on mindfulness and cognitive domains of parenting were significant, the impacts on parenting practice constructs were relatively smaller. Future digital health research needs to further investigate strategies to support parenting practice change. *Three*, our evaluation study relied on parental and CHW reported data, which might have reporter bias. Future studies should consider objective observation measures.

## Conclusions

This is the first study to test whether a screening, brief intervention, and referral to treatment (SBIRT) prevention service model can be applied in FBOs in LMICs to promote early behavioral health screening, parents’ pediatric behavioral health literacy, and children’s behavioral health outcomes. To consider implementation resource and health access gaps, implementation of the SBIRT was guided by a digital health approach and implemented by trained CHWs, with support from FBO leaders. Findings of this study support the feasibility of implementing a CHW implemented digital health program for promoting pediatric behavioral health in FBOs, and simultaneously improving preventive service access and strengthening the community mental health system for public health. To ensure the digital solution was engaging, we co-designed the P-BHDT with local expert and iteratively adapted based on parents and caregivers as well as CHWs and experts’ feedback and suggestions (in content, functions, and delivery model) through a series of focus groups and design thinking workshops. For parents, we considered strategies to address parents’ education, digital health literacy, and trust factors (i.e., including providing CHW support, FBO leaderships endorsement). For implementers (CHWs), we considered their digital readiness and usage patterns when designing the implementation procedure and training program (i.e., assessing CHWs’ digital readiness to promote awareness, adding digital literacy training). Lessons learned from this study can be applied to implementation of similar public health approach of digital health intervention in other low-income countries or low behavioral health resource communities.

## Figures and Tables

**Table 1:** summarizes the demographic characteristics of our recruited FBOs, CHWs, and families.

	Total (n=218 parents) (n=21 CHWs)	Intervention (n=91 parents) (n = 10 CHWs)	Control (n = 127 parents) (n=11 CHWs)	*p*
	
*FBO*	Mean (SD) / %	Mean (SD) / %	Mean (SD) / %	
	
Average # of congregation members	300 (198.16)	275 (289.91)	325 (176.78)	.854
Average # of children at home (aged 3–8)	122.5 (98.45)	145 (148.49)	100 (70.71)	.368
Has children group	100%	100%	100%	-

***CHWs Characteristics***				
Year in FBO	11.04 (10.30)	12.29 (12.47)	9.79 (8.40)	.668
Age				
20–39	76.2%	60.0%	90.9%	.097
40–59	23.8%	40.0%	9.1%	
Marital Status- Single	63.2%	44.4%	80.0%	.165
Sex-Female	61.9%	60.0%	63.6%	.864
Education				
Secondary	14.3%	30.0%	0.0%	.060
Tertiary/college	57.1%	60.0%	54.5%	
More than college	28.6%	10.0%	45.5%	
Have children	52.4%	70.0%	36.4%	.198
Involve in child health promotion before	15.8%	20.0%	11.1%	.596
Own mobile phone	100.0%	100.0%	100.0%	-
Own a smart phone	81.0%	90.0%	72.7%	.314
Technology Readiness (1–5)	4.19 (.41)	4.11 (.40)	4.26 (.41)	.436

***Family Demographic***				
Parent sex- Female	82.5%	86.8%	79.4%	.205
Relationship				
Mother	60.3%	56.0%	63.6%	.439
Father	13.9%	13.2%	14.4%	
Grandparent	20.1%	25.3%	16.1%	
Other	5.7%	5.5%	5.9%	
Parent Education				
Primary or less	29.6%	30.8%	28.8%	.929
Secondary	34.3%	33.0%	35.2%	
>= High school	36.1%	36.3%	36.0%	
Marital Status				
Married	46.4%	50.5%	43.3%	.190
Live with partner	23.7%	17.6%	28.3%	
Single/Widow/ Divorce	29.9%	31.9%	28.3%	
Language- English	38.2%	44.0%	34.1%	.142
Employed	70.4%	72.1%	67.3%	.563
Food insecure	18.8%	20.0%	17.9%	.661
Child Sex- Male	46.0%	45.0%	47.6%	.678
Child age	5.41 (1.68)	5.38 (1.66)	5.43 (1.69)	.830

SD: Standard Deviation; FBO: Faith-based organizations; CHW: Community Health Worker

***Note.*** Randomization was on the FBO level.

**Table 2.** Digital Toolkit Implementation Outcomes Measures and Results

Outcome Indicators	Items (score range)	α	Sample Items	Mean (SD)
***Acceptability, Appropriateness, and Usefulness***				
Acceptability (P/ CHW)	4 (1–5)	.68/.71	• The Digital Toolkit Program meets my expectation • I welcome the Digital Toolkit Program in our church	P: 4.42 (.49) CHW: 4.7 (.33)
Appropriateness (P/ CHW)	4 (1–5)	.73/.95	• The Digital Toolkit Program fits my/our community needs • The program is applicable to our parents and faith-based members	P: 4.39 (.47) CHW: 4.55 (.51)
Parent Perceive Usefulness (P)	7 (1–5)	.75	• The interview questions and report card helped me and my family better understand and support my child • The parenting strategies generated from the tablet based on my responses is useful and relevant to my needs • The report card results reflect what my child and my family like	P: 4.33 (.44)
Perceive Usefulness for Parents (CHW)	4 (1–4)	.95	• The Digital Toolkit program is a good way to help parents learn about their child’s and family’s strengths and weakness • Parents that I have interviewed using the Digital Toolkit were happy with the time spent and fount it useful	P: 3.75 (.35)
***Implementation Feasibility (Users’ comfort, trust to the Digital Toolkit & Implemented’ experience)***				
Trust in data security and safety (P)	2 (1–5)	.79	• Trust my data on the Tablet are confidential, just between the interviewer and me • Trust the security in place for the tablet questionnaire	P: 4.28 (.65)
Comfortable in Answering Personal Questions (P)	3 (1–5)	.71	• Feel comfortable in discussing my child’s behavioral, developmental, and learning challenges with the CHW • Feel comfortable in discussing family issues and other family problems with the CHW	P: 4.10 (.96)
Engaging FBO is a Feasible Strategy to Promote Participation (P)	2 (1–5)	.66	• Church leaders’ support to the Digital Program make me more comfortable and interested in signing up the interview session • Many parents from my church have signed up the session, which make me more comfortable to share parenting experience and challenges	P: 4.21 (.62)
Feasibility to Implement (CHW)	4 (1–5)	.74	• The Digital Toolkit Program is Implementable • The Digital Toolkit Program is easy to use and implement	CHW: 4.45 (.74)
***FBO CFIWs’ Ability in Implementing P-BHDT***				
CHW Competency (P)	10 (1–5)	.88	• When sharing the Report, the CHW gave me time to reflect and asked me to share my view • The CHW explains things clearly • The CHW seems skilled and have no trouble in using the Digital Toolkit • I feel the CHW pays attention on what I said and understands me	P: 4.40 (.40)

P: Parent reported data; CHW: Community health workers P-BHDT: Pediatric-Behavioral Health Digital Tool.

***Note.*** Implementation data were gathered from parents and CHWs 2–3 months after implementing the P-BHDT program.

**Tabie 3.** Preliminary Intervention Effectiveness on Parenting and Child Outcomes

			Baseline				Intervention Effect		

Parenting Outcomes	Items (score range)	α	Total (n=218 parents)	Intervention (n=91 parents)	Control (n=127 parents)	p	Pre-Post Differences (Model-based Mean Difference)	Effect Size (d)	p

Perceive Effectiveness in Discipline	2 (1–5)	0.66	3.98 (.69)	3.85 (.60)	4.07 (.74)	0.021	.89 (.28)	1.29	.003
Setting Rules/Routine	4 (1–5)	0.69	4.02 (.77)	4.08 (.70)	3.97 (.82)	0.266	.24 (.22)	0.31	.137
Nurturing parenting	9 (1–5)	0.78	3.67 (.64)	3.77 (.54)	3.64 (.60)	0.138	.17 (.11)	0.27	.270
Mindfulness parenting	10 (1–4)	0.84	2.94 (.61)	2.90 (.62)	2.96 (.61)	0.499	.98 (.45)	1.61	.049
P-Mental Health (PHQ4)	4 (0–12)	0.76	2.61 (2.81)	2.69 (2.67)	2.56 (2.92)	0.731	2.47 (.96)	0.88	.018

**Child Social Emotional Outcomes**									
Child Emotion Regulation	6 (−4)	0.78	2.27 (.90)	2.42 (.85)	2.17 (.92)	0.036	.98 (.45)	1.09	.049
Parent-Child Conflict Relationship	5 (1–5)	0.68	1.75 (.73)	1.96 (.67)	1.60 (.73)	<.001	−.79 (.25)	−1.08	.002

***Note,* α** is internal consistency forthe scale based on the entire study sample. Preliminary Effectiveness findings in table were estimated based on the imputed data (accounting for missingness), with a total N of 91. Imputed and non-imputed findings yield consistent results. About 16.5% parents were at-risk for mental health problems (PHQ4), with no baseline group difference between intervention and control.
